# Efficacy and safety of Qingre Lishi decoction for type 2 diabetes: a systematic review and meta-analysis

**DOI:** 10.3389/fendo.2025.1604633

**Published:** 2025-10-20

**Authors:** Zimei Lin, Yi Sun, Juan Li, Likun Du

**Affiliations:** ^1^ The First Clinical Medical College, Heilongjiang University of Chinese Medicine, Harbin, Heilongjiang, China; ^2^ Department of Endocrinology II, The First Affiliated Hospital of Heilongjiang University of Chinese Medicine, Harbin, Heilongjiang, China

**Keywords:** efficacy, Qingre Lishi decoction, type 2 diabetes, systematic review, meta-analysis

## Abstract

**Objective:**

This systematic review and meta-analysis aimed to evaluate the efficacy and safety of Qingre Lishi decoction (QRLSD) for type 2 diabetes (T2D), thereby providing new evidence-based insights into the role of traditional Chinese medicine in treating T2D.

**Methods:**

WanFang, Embase, Web of Science, PubMed, China National Knowledge Infrastructure, and the Cochrane Library were searched up to January 2025. Studies assessing the efficacy and safety of QRLSD in treating T2D were included. Data were pooled using standardized mean difference (SMD) and risk ratio (RR). Sensitivity and subgroup analyses were performed to assess the stability of the results and investigate potential sources of heterogeneity. Statistical analyses were conducted using RevMan 5.4 and Stata 18.0.

**Results:**

A total of 18 randomized controlled trials (RCTs) were included. Our results showed that QRLSD reduced the levels of fasting blood glucose (SMD: -15.09; 95% CI: [-18.27, -11.90], I² = 99%, P < 0.00001), 2-hour postprandial blood glucose (SMD: -17.71; 95% CI: [-21.70, -13.71], I² = 98%, P < 0.00001), glycated hemoglobin (SMD: -18.93; 95% CI: [-22.77, -15.09], I² = 98%, P < 0.00001), as well as other metabolic parameters, indicators of insulin resistance, and body mass index. No significant difference in the incidence of adverse events between the intervention and control groups was found (RR: 1.8; 95% CI: [0.57, 5.68], I² = 9%, P=0.32).

**Conclusion:**

For T2D, QRLSD can significantly improve clinical outcomes and may serve as a more effective therapy than single Western medicine therapy. Moreover, it was not related to significant adverse events. Given the inherent limitations of this study, large-scale, multicenter international RCTs are needed to further validate these findings in the future.

**Systematic review registration:**

https://www.crd.york.ac.uk/prospero/, identifier CRD420251000585.

## 1 Introduction

Diabetes is a chronic metabolic disease with a rising prevalence worldwide. In China, the prevalence of diabetes increased from only 0.67% in 1980 to 12.4% by 2010 (1). This trend is mirrored globally: by 2021, approximately 537 million people were affected by diabetes, corresponding to a global prevalence of 6.1% (1). The number is expected to continue rising, with global prevalence projected to reach 10% by 2050, and no country is expected to experience a decline (2). The majority of new cases are from low- and middle-income countries, posing substantial challenges to their healthcare systems and public finances. Diabetes ranks as the eighth leading cause of death and disability worldwide (3). In China, type 2 diabetes (T2D) remains the predominant form of the disease, with its high prevalence driven by urbanization, lifestyle changes, overweight and obesity, as well as genetic susceptibility (4). Given the serious health and societal burdens posed by diabetes both globally and domestically, it remains a critical public health concern requiring continued attention and the development of novel solutions.

According to the Prevention or Delay of Diabetes and Associated Comorbidities: Standards of Care in Diabetes—2025 (5), current treatment strategies for T2D include lifestyle interventions, weight management, insulin injection, and metformin, along with newer pharmacological agents such as GLP-1 receptor agonists, SGLT-2 inhibitors, and DPP-4 inhibitors.

However, conventional treatments are associated with various adverse effects. For instance, SGLT-2 inhibitors are related to genitourinary infections, fournier gangrene, lower-limb amputations, euglycemic diabetic ketoacidosis, fractures, and orthostatic hypotension; GLP-1 receptor agonists may elevate the risk of pancreatitis, acute cholecystitis, and diabetic retinopathy; and DPP-4 inhibitors might elevate the risk of chronic heart failure (6). When the levels of blood glucose are extremely high, insulin injection is typically recommended for treatment. The latest medications, such as once-weekly insulin icodec and basal insulin BIF, are in clinical development. Nevertheless, challenges including poor patient adherence, increased insulin resistance, and high costs remain unresolved (7). Thus, it is important to explore new therapies for T2D. Diabetes has long been studied in Traditional Chinese medicine (TCM), and it is defined as an emaciation-thirst disease. Modern TCM practitioners often correlate its pathogenesis with damp-heat syndrome (8). Some researchers suggest that T2D is frequently accompanied by insulin resistance, which is closely linked to inflammation. Qingre Lishi decoction (QRLSD) can improve the prognosis of T2D by modulating inflammatory levels, thus serving as a prospective treatment option (9). In this study, the Qingre Lishi decoction (QRLSD) included only those formulas explicitly indicated in treatment principles to have heat-clearing and dampness-draining effects. The constituent herbs in these formulas were broadly categorized as either heat-clearing or dampness-draining. Due to the complex naming conventions of TCM formulas, some classical decoctions are named after their main herb or the original targeted effect. For example, in Qingxin Lianzi Yin decoction, “Qingxin” indicates its function of clearing heart heat, while “Lianzi” refers to its main active ingredient, Nelumbo nucifera Gaertn. A similar example is Qinlian decoction combined with Pingwei San. Other formulas, such as Yinchen Wuling San, Gegen Qinlian decoction (GQD), and Yinchenhao decoction (YCHD), are named directly after their primary ingredients. Given the long history of TCM, classical formulas are sometimes repurposed for modern indications, and certain traditional formulas exhibit clear efficacy for such diseases as T2D. Additionally, some ancient formulas and modern formulations created by contemporary practitioners are directly named according to their therapeutic effects, such as the Qingre Lishi formula, with occasional repetition of names (10). Regardless of naming, all included formulas were explicitly reported by the investigators to exert heat-clearing and dampness-draining effects. The aforementioned GQD and YCHD are examples of formulas that fall within this category. Studies have found that GQD can significantly lower the levels of fasting blood glucose (FBG), as well as alleviate ectopic fat deposition, oxidative stress, and inflammation in the liver tissue of obese rats (11). Other studies have demonstrated that YCHD can suppress cellular inflammatory responses by modulating inflammatory signaling pathways. Thus, it is effective in treating diabetes and its complications (12).

Although a large number of relevant studies have been published, most are limited to small-sample clinical or non-clinical trials conducted at single centers. TCM decoctions are highly diverse, and even among formulas with heat-clearing and dampness-eliminating effects, their names vary. For example, GQD and YCHD have not been systematically categorized. Moreover, outcome measures differ widely, focusing either on changes in serum levels or on improvements in physical indicators. Furthermore, the overall level of evidence is low, primarily based on clinical observations, lacking data linking results to the broader category of heat-clearing and dampness-eliminating treatments (13). In addition, evidence supporting the efficacy of QRLSD in treating diabetes is scarce, and no systematic evaluation of its effectiveness has been conducted. Therefore, a systematic analysis and assessment of the efficacy and safety of QRLSD for the treatment of T2D is warranted.

## 2 Materials and methods

### 2.1 Protocol and registration

This study was conducted in accordance with the PRISMA (Preferred Reporting Items for Systematic Reviews and Meta-Analysis) 2020 statement released by Cochrane (14) and was registered in the PROSPERO database (CRD420251000585).

### 2.2 Search methods

WanFang, PubMed, the Cochrane Library, Embase, Web of Science, and China National Knowledge Infrastructure were searched up to February 2025 to select randomized controlled trials (RCTs) and cohort studies assessing the efficacy and safety of QRLSD in treating T2D. The Chinese search terms included Qingre Lishia decoction and diabetes, while the English search terms were Type 2 Diabetes & T2DM and Qingrelishi & Qingre Lishia. There were no language or geographical restrictions. Additionally, references to the selected studies were reviewed for additional eligible studies. The search strategy is presented in **Supplementary File 1**.

### 2.3 Inclusion and exclusion criteria

Eligible RCTs and cohort studies were selected after reviewing titles, abstracts, and full texts according to the following criteria.

The inclusion criteria were as follows: (i) population: adults (aged ≧̸18 years) clinically diagnosed with T2D, with no restrictions on sex or race; (ii) intervention and comparison: the control group received Western medicine therapy and/or lifestyle interventions or other treatments. The intervention group received either QRLSD alone or QRLSD in combination with the treatment administered to the control group; (iii) outcomes: studies included at least one of the following outcomes: primary outcomes included clinical efficacy and the incidence of adverse events; secondary outcomes included the levels of FBG, 2-hour postprandial blood glucose (2hPG), and glycated hemoglobin (HbA1c).

The exclusion criteria were as follows: (i) case-control studies, single-arm trials, review articles, animal studies, and cross-sectional studies; (ii) participants with elevated levels of blood glucose caused by other conditions; (iii) intervention groups received other types of TCM therapy (Chinese medicine pills, massage, acupuncture, injections, Chinese patent medicine, or auricular acupuncture); (iv) studies with inaccurate data or incomplete outcome measurements, or with inaccessible data from the original authors; (v) duplicate publications.

### 2.4 Data extraction

Two reviewers (Zimei Lin and Yi Sun) independently extracted data based on the search strategy and inclusion criteria. The extracted data included: (i) publication information: publication year, first author, and title; (ii) study characteristics: study design and duration of the intervention; (iii) participant characteristics: age, sex distribution, number of participants, average FBG, average age, and average BMI; (iv) intervention: medication, frequency, dosage, and route of administration for the control group; dosage, the composition of the TCM formula, and frequency for the intervention group; (v) outcomes (primary and secondary outcomes): continuous data were expressed as means or standard deviations, and categorical data were expressed as event counts or total numbers. Disagreements were resolved by a third reviewer (Juan Li).

### 2.5 Quality assessment

The Cochrane Risk of Bias (RoB) tool was utilized to evaluate the quality of included RCTs by two reviewers (Zimei Lin and Yi Sun) (15). Moreover, they cross-examined the evaluation results. The assessment encompassed seven domains: allocation concealment, blinding of participants and personnel, reporting bias, incomplete outcome data, random sequence generation, blinding of outcome assessment, and other bias. The methodological quality of the eligible studies was categorized as low, high, or unclear. The Newcastle-Ottawa Scale (NOS) was applied to evaluate the quality of the included cohort, with 7–9 points indicating high quality (16). Any disagreements were resolved by a third reviewer (Juan Li).

### 2.6 Statistical analysis

Literature management was carried out using EndNote X9, and data were organized in Excel. Statistical analyses were conducted through RevMan 5.4 and Stata 18.0. For categorical variables, effect sizes were presented as risk ratios (RR) with 95% confidence intervals (CI). For continuous variables, effect sizes were expressed as mean differences (MD) or standardized mean differences (SMD) with 95% CIs. Heterogeneity was assessed using the Cochrane Q test and the I² statistic. If P < 0.05 and I² ≥ 50%, which suggested significant heterogeneity, a random-effects model was applied for all analyses. Sensitivity and subgroup analyses were carried out to investigate the stability of the results and sources of heterogeneity. The funnel plots and Egger’s test were used to assess publication bias, with a significance level of P < 0.05. Additionally, according to GRADE, the quality of evidence for each outcome was assessed and graded as “high”, “moderate”, “low”, or “very low” to draw conclusions (17).

## 3 Results

### 3.1 Research and selection

**Figure 1:**
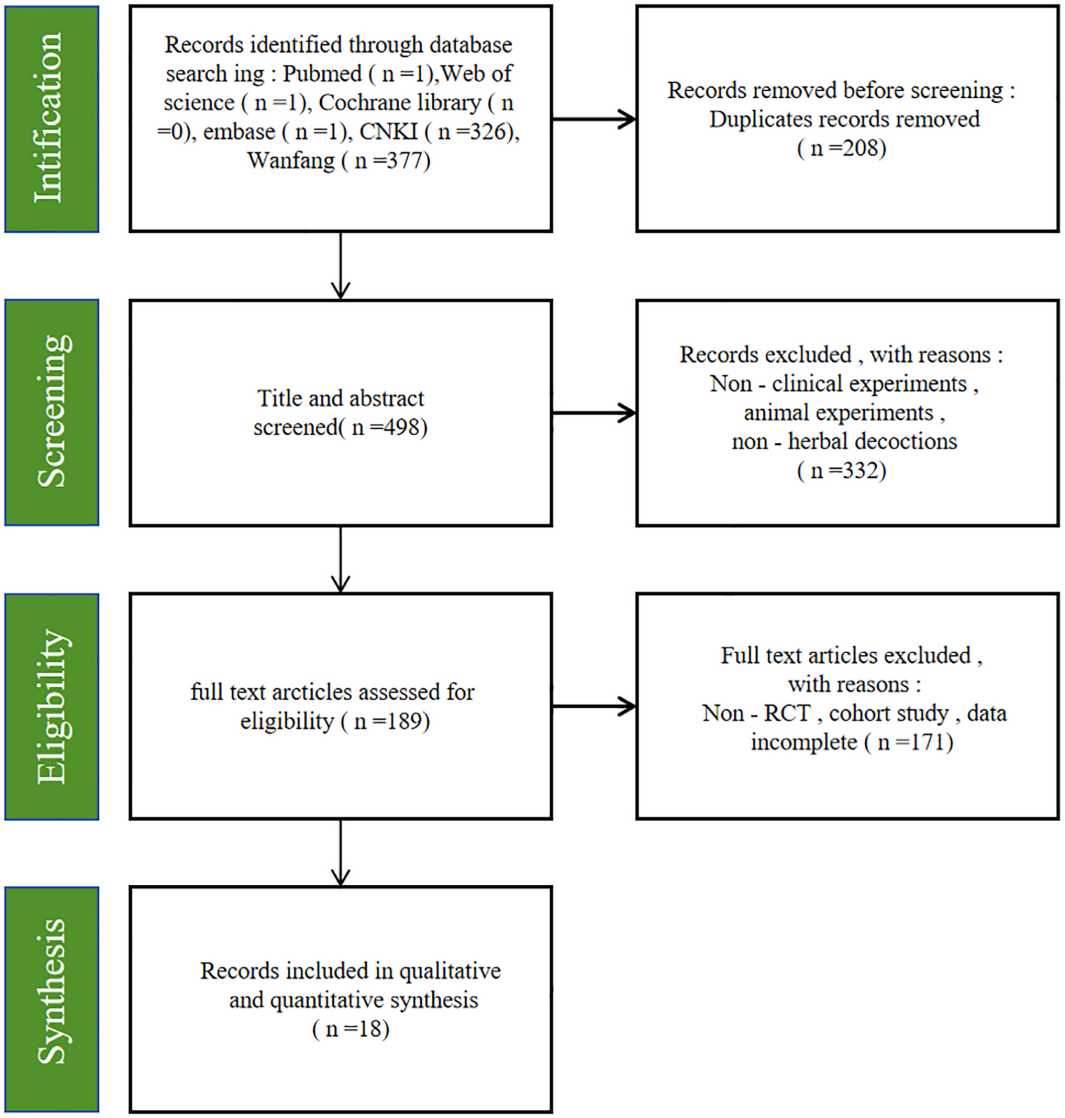
Flow plot.

A total of 706 relevant studies were identified from the databases. After removing 208 duplicate records, 498 studies remained. Subsequently, 332 studies were further removed after reviewing titles and abstracts. A total of 189 studies remained for full-text review. Finally, 18 studies were included in this study (18–35). **Figure 1** illustrates the selection process, and **Table 1** provides the basic characteristics of the eligible studies.

**Table 1:** Basic characteristics of the included studies.

Study	Region	Study design	Intervention	Control	Patients (E/C)	Treatment time (weeks)	Mean age (E/C) (year)	Male (E/C)	Female (E/C)	Mean BMI (E/C) (kg/m²)	Mean FBG (E/C) (mmol/L)
Duan 2012 (18)	China	RCT	Qingxin Lianzi Decoction	Metformin	37/37	12	57.75/ 56.25	15/18	17/21	24.44 ± 1.44/ 25.13 ± 1.73	9.35 ± 1.23/ 8.99 ± 0.94
Fu 2016 (19)	China	RCT	Gegen QinlianXiaoke Decoction+Metformin	Metformin	40/41	8	—/—	23/20	—/—	—/—	—/—
Gao 2004 (20)	China	RCT	Qingre Lishi Decoction+Diamicron	Diamicron	40/30	4	—/—	—/—	26/27	—/—	9.33 ± 1.02/ 9.62 ± 0.93
Niu 2020 (21)	China	RCT	Jianpi Qingre Lishi Decoction+Metformin	Metformin	37/37	12	67.27 ± 4.44/ 65.89 ± 3.84	11/10	18/17	30.09/ 30.44	9.35 ± 2.14/ 9.47 ± 1.58
Qin 2015 (22)	China	RCT	Yinchenhao Decoction+Metformin	Metformin	30/27	8	60 ± 6.32/ 59.79 ± 7.44	12/10	26/25	—/—	7.917 ± 2.117/7.743 ± 2.667
Wang 2006 (23)	China	RCT	Qingre Lishi Decoction+Metformin	Metformin	50/46	12	53.7 ± 3.67/ 52.93 ± 3.95	24/21	14/17	23.73 ± 2.133/23.04 ± 1.906	11.38 ± 2.05/ 11.41 ± 2.14
Wei 2005 (24)	China	RCT	Yinchen Wuling Decoction+Diamicron	Metformin	40/40	8	44.06 ± 9.55/ 45.94 ± 10.38	26/23	17/13	28.3 ± 4.51/ 28.9 ± 5.03	8.4 ± 0.53/ 8.82 ± 0.46
Wei 2011 (25)	China	RCT	Qingre Lishi Decoction+Diamicron, Diamicron	Metformin+ Diamicron	30/30	8	53.22 ± 10.82/ 53.62 ± 9.98	16/14	15/12	25.98 ± 1.63/ 25.85 ± 1.52	8.96 ± 1.91/ 10.08 ± 2.96
Wei 2024 (26)	China	cohort study	Qingre Lishi Decoction	Lifestyle adjustment	35/35	4	45.77 ± 11.36/ 49.59 ± 12.09	16/10	26/26	—/—	—/—
Xu 2022 (27)	China	RCT	Qingre Lishi Decoction+Insulin	Insulin	52/52	2	59.98 ± 11.03/ 61.09 ± 10.34	28/24	19/20	24.01 ± 2.76/ 24.63 ± 2.92	8.81 ± 0.54/ 8.87 ± 0.58
Xuan 2021 (28)	China	RCT	Jianpi Qingre Lishi Decoction+Metformin	Metformin	35/35	8	54.46 ± 5.8/ 53.31 ± 6.06	16/15	25/26	28.36 ± 0.23/ 28.46 ± 0.27	11.53 ± 2.46/ 11.55 ± 2.43
Yu 2020 (29)	China	RCT	Jianpi Qingre Lishi Decoction+Metformin	Metformin	48/48	12	53.48 ± 7.8/ 53.21 ± 8.2	23/22	29/33	—/—	9.67 ± 0.24/ 9.93 ± 1.37
Yu 2023 (30)	China	RCT	Qingre lishi Hutan Decoction+Dapagliflozin	Dapagliflozin	75/75	24	62.31 ± 7.5/ 60/9 ± 6.82	46/42	11/14	32.24 ± 5.39/ 30.98 ± 5.71	8.65 ± 2.16/ 8.67 ± 2.09
Zhang 2011 (31)	China	RCT	Qinlian Pingwei Decoction	Metformin	36/36	8	55.1 ± 14.3/ 53.4 ± 5.2	25/22	16/18	—/—	6.24 ± 0.37/ 6.33 ± 0.4
Zhang 2015 (32)	China	RCT	Jianpi Qingre Lishi Decoction	Metformin	30/30	12	52.13 ± 8.1/ 52.82 ± 7.68	14/12	12/11	26.5 ± 1.35/ 26.7 ± 1.75	7.87 ± 2.03/ 8.07 ± 1.73
Zhang 2016 (33)	China	RCT	Qingre Lishi Decoction	Lifestyle adjustment	34/32	4	51 ± 17/ 49 ± 16	22/21	20/18	26.31 ± 1.45/ 26.25 ± 1.51	9.26 ± 1.52/ 9.43 ± 1.38
Zhang 2023 (34)	China	RCT	Jianpi Qingre Lishi Decoction+Metformin	Metformin	42/42	12	44.25 ± 7.1/ 46.08 ± 8.42	24/26	10/13	28.12 ± 1.89/ 27.87 ± 2.02	9.62 ± 1.78/ 9.4 ± 1.29
Zhi 2024 (35)	China	RCT	Jianpi Qingre Lishi Decoction+Metformin, GLP-1	Metformin	25/30	12	61.35 ± 8.8/ 61.35 ± 3.63	15/17	17/21	29.37 ± 1.21/ 29.84 ± 1.21	9.35 ± 1.23/ 8.99 ± 0.94

### 3.2 Study characteristics

The 18 eligible studies involved 1419 individuals, with 716 in the intervention group and 703 in the control group. The sample sizes in both groups exceeded 25 cases. All included patients were from China. The mean age varied from 44.06 to 67.27 years in the intervention cohort and 45.94 to 65.89 years in the control cohort. The duration of the intervention in all studies was more than two weeks. The control group received conventional treatment, including insulin, metformin, sulfonylureas, and GLP-1 agonists. The intervention group received either QRLSD alone or in combination with the treatments administered to the control group. The QRLSD included YCHD, GQD, and customized formulas with commonly used herbs such as Qinghao (herba artemisiae scopariae), Fuling [wolfiporia cocos (F.A. Wolf) ryvarden and gilb], Gegen (radix puerariae), and Huanglian (coptis chinensis Franch.) (as illustrated in **Table 2**).

**Table 2:** Ingredients of the prescription.

Study	Intervention	The composition of the prescription
Duan 2012 (18)	Qingxin Lianzi Decoction	Huangqi (Astragalus membranaceus (Fisch.) Bunge) 30g; Dangshen (Codonopsis pilosula (Franch.) Nannf.) 30g; Fuling (Poria cocos (F.A. Wolf) Ryvarden & Gilb.) 15g; Baizhu (Atractylodes macrocephala Koidz.) 15g; Lianzi (Nelumbo nucifera Gaertn.) 15g; Maidong (Ophiopogon japonicus (L. f.) Ker Gawl.) 15g; Huangqin (Scutellaria baicalensis Georgi) 15g; Gouqizi (Lycium barbarum L.) 15g; Cheqianzi (Plantago asiatica L.) 15g; Gancao (Glycyrrhiza uralensis Fisch.) 6g.
Fu 2016 (19)	Gegen QinlianXiaoke Decoction	Gegen (Radix Puerariae) 30g; Huangqin (Scutellaria baicalensis Georgi) 15g; Huanglian (Coptis chinensis Franch.) 10g; Gancao (Glycyrrhiza uralensis Fisch.) 6g; Huangqi (Astragalus membranaceus (Fisch.) Bge.) 20g; Dangshen (Radix Codonopsitis) 30g; Baizhu (Atractylodes macrocephala Koidz.) 15g; Fuling (Poria cocos (Schw.) Wolf) 20g; Zexie (Alismatis Rhizoma) 10g; Zhuling (Polyporus umbellatus (Pers.) Fries) 10g; Yumixu (Stigma Maydis) 10g; Rougui (Cortex Cinnamomi Cassiae) 10g; Danshen (Salvia miltiorrhiza Bunge) 20g; Shengjiang (Rhizoma Zingiberis) 10g; Dihuang (Rehmannia glutinosa (Gaertn.) Libosch.) 20g.
Gao 2004 (20)	Qingre Lishi Decoction	Huangqin (Scutellaria baicalensis Georgi) 12g; Huanglian (Coptis chinensis Franch.) 6g; Qinghao (Herba Artemisiae Scopariae) 25g; Jinyinhua (Lonicera japonica Thunb.) 15g; Lianqiao (Forsythia suspensa (Thunb.) Vahl) 10g; Pugongying (Taraxacum mongolicum Hand.-Mazz.) 30g; Huashi (Talcum) 20g; Gualougen (Radix Trichosanthis) 20g; Shanyao (Rhizoma Dioscoreae) 30g; Zisu (Agastache rugosa (Fisch. et Mey.) O. Ktze.) 9g; Roudoukou (Myristica fragrans Houtt.) 6g.
Niu 2020 (21)	Jianpi Qingre Lishi Decoction	Taizishen (Radix Pseudostellariae) 20g; Shanyao (Rhizoma Dioscoreae) 20g; Cangzhu (Atractylodes lancea (Thunb.) DC.) 15g; Xuanshen (Scrophularia ningpoensis Hemsl.) 15g; Gegen (Radix Puerariae) 30g; Huangqin (Scutellaria baicalensis Georgi) 15g; Huanglian (Coptis chinensis Franch.) 15g; Fanbaicao (Potentilla discolor) 30g; Guijianyu (Euonymus alatus (Thunb.) Sieb.) 30g; Lizhihe (Semen Litchi) 30g; Gancao (Glycyrrhiza uralensis Fisch.) 10g.
Qin 2015 (22)	Yinchenhao Decoction	Qinghao (Herba Artemisiae Scopariae) 30g; Dahuang (Rheum officinale Baill.) 6g; Houpo (Magnolia officinalis Rehd. et Wils.) 12g; Huangqi (Astragalus membranaceus (Fisch.) Bunge) 30g; Banxia (Pinellia ternata (Thunb.) Breit.) 9g; Cangzhu (Atractylodes lancea (Thunb.) DC.) 12g.
Wang 2006 (23)	Qingre Lishi Decoction	Cangzhu (Atractylodes lancea (Thunb.) DC.) 15g; Huanglian (Coptis chinensis Franch.) 6g; Qinghao (Herba Artemisiae Scopariae) 30g; Zelan (Eupatorium fortunei Turcz.) 15g; Zhiqiao (Fructus Aurantii) 15g; Dijincao (Euphorbia humifusa Willd.) 30g; Houpo (Magnolia officinalis Rehd. et Wils.) 10g; Jineijin (Endothelium Corneum Gigeriae Galli) 15g.
Wei 2005 (24)	Yinchen Wuling Decoction	Qinghao (Herba Artemisiae Scopariae) 25g; Fuling (Wolfiporia cocos (F.A. Wolf) Ryvarden & Gilb.) 20g; Zexie (Alisma plantago-aquatica Linn.) 15g; Baizhu (Atractylodes macrocephala Koidz.) 12g; Zhuling (Polyporus umbellatus (Pers.) Fr.) 15g; Rougui (Cinnamomum cassia Presl.) 6g; Yumixu (Stigma Maydis) 20g; Lingzhi (Ganoderma lucidum (Leyss. ex Fr.) Karst.) 20g; Sanqi (Panax pseudo-ginseng Wall. var. notoginseng (Burkill) Hoo & Tseng.) 10g.
Wei 2011 (25)	Qingre Lishi Decoction	Huanglian (Coptis chinensis Franch.) 10g; Huangbai (Phellodendron amurense Rupr.) 10g; Sangbaipi (Cortex Morus Alba) 15g; Zexie (Alisma plantago-aquatica Linn.) 12g; Gegen (Radix Puerariae) 15g; Cangzhu (Atractylodes lancea (Thunb.) DC.) 10g; Shanzha (Crataegus pinnatifida Bge.) 20g.
Wei 2024 (26)	Qingre Lishi Decoction	Chaihu (Radix Bupleuri) 15g; Fabanxia (Rhizoma Pinelliae Praeparatum) 15g; Huangqin (Scutellaria baicalensis Georgi) 15g; Jiudahuang (Rheum officinale Baill. processed with wine) 15g; Zhuru (Sinocalamus affinis) 15g; Zhishi (Fructus Aurantii Immaturus) 10g; Zhimu (Anemarrhena asphodeloides) 10g; Shengshigao (Gypsum Fibrosum) 15g; Huanglian (Coptis chinensis Franch.) 15g; Guizhi (Ramulus Cinnamomi Cassiae) 10g; Ganjiang (Rhizoma Zingiberis) 10g; Wumei (Fructus Mume) 5g; Wuweizi (Schisandra chinensis (Turcz.) Baill.) 5g.
Xu 2022 (27)	Qingre Lishi Decoction	Yimucao (Leonurus japonicus Houtt.) 30g; Yiyiren (Semen Coicis, Coix lachryma-jobi L. var. ma-yuen (Roman.) Stapf) 30g; Tufuling (Smilax glabra Roxb.) 15g; Heye (Folium Nelumbinis) 15g; Zhimu (Anemarrhena asphodeloides Bunge) 15g; Chenpi (Dried Tangerine Peel, Citrus reticulata Blanco) 15g; Danshen (Salvia miltiorrhiza Bunge) 15g; Tiannanxing (Arisaema heterophyllum Blume) 15g; Cangzhu (Atractylodes lancea (Thunb.) DC.) 12g; Zexie (Alisma orientale (Sam.) Juz.) 12g; Mudanpi (Cortex Moutan, Paeonia suffruticosa Andr.) 12g; Dihuang (Rehmannia glutinosa (Gaertn.) Libosch.) 12g; Shanyao (Rhizoma Dioscoreae, Dioscorea opposite Thunb.) 12g; Niuxi (Achyranthes bidentata Blume) 12g; Shanzhuyu (Cornus officinalis Sieb. et Zucc.) 12g; Huanglian (Coptis chinensis Franch.) 10g; Banxia (Pinellia ternata (Thunb.) Breit.) 10g; Chuanbeimu (Fritillaria cirrhosa D.Don) 6g.
Xuan 2021 (28)	Jianpi Qingre Lishi Decoction	Gegen (Radix Puerariae) 30g; Huanglian (Coptis chinensis Franch.) 9g; Huangqin (Scutellaria baicalensis Georgi) 9g; Banxia (Pinellia ternata (Thunb.) Breit.) 9g; Gancao (Glycyrrhiza uralensis Fisch.) 6g; Chenpi (Citrus sinensis (L.) Osbeck) 12g; Shanyao (Rhizoma Dioscoreae) 30g; Shanzha (Crataegus pinnatifida Bge.) 30g; Baizhu (Atractylodes macrocephala Koidz.) 15g; Gualou (Fructus Trichosanthis) 15g; Shengjiang (Zingiber officinale Rosc.) 3g.
Yu 2020 (29)	Jianpi Qingre Lishi Decoction	Huanglian (Coptis chinensis Franch.) 10g; Shengjiang (Rhizoma Zingiberis) 3g; Chuanxiong (Ligusticum chuanxiong Hort.) 10g; Baishao (Radix Paeoniae Alba) 20g; Yiyiren (Semen Coicis, Coix lachryma-jobi L. var. ma-yuen (Roman.) Stapf) 30g; Tufuling (Poria cocos (Schw.) Wolf) 15g; Jiaogulan (Gynostema pentaphyllum (Thunb.) Makino) 20g; Tufuling (Smilax glabra Roxb.) 30g; Taizishen (Radix Pseudostellariae) 10g; Shanyao (Rhizoma Dioscoreae) 15g; Dihuang (Rehmannia glutinosa (Gaertn.) Libosch.) 15g; Guijianyu (Euonymus alatus (Thunb.) Sieb.) 30g; Shijueming (Concha Haliotidis) 15g; Danshen (Salvia miltiorrhiza Bunge) 30g; Sangye (Folium Mori) 10g; Gancao (Glycyrrhiza uralensis Fisch.) 3g.
Yu 2023 (30)	Qingre lishi Hutan Decoction	Yimucao (Leonurus japonicus Houtt.) 30g; Tufuling (Smilax glabra Roxb.) 15g; Yiyiren (Semen Coicis, Coix lachryma-jobi L. var. ma-yuen (Roman.) Stapf) 30g; Heye (Folium Nelumbinis) 15g; Chenpi (Citri Reticulatae Pericarpium) 15g; Zhimu (Anemarrhena asphodeloides Bunge) 15g; Danshen (Salvia miltiorrhiza Bunge) 15g; Cangzhu (Atractylodes lancea (Thunb.) DC.) 12g; Tiannanxing (Arisaema heterophyllum Blume) 15g; Zexie (Alisma orientale (Sam.) Juz.) 12g; Dihuang (Rehmannia glutinosa (Gaertn.) Libosch.) 12g; Mudanpi (Cortex Moutan, Paeonia suffruticosa Andr.) 12g; Shanyao (Rhizoma Dioscoreae) 12g; Niuxi (Achyranthes bidentata Blume) 12g; Shanzhuyu (Cornus officinalis Sieb. et Zucc.) 12g; Huanglian (Coptis chinensis Franch.) 10g; Chuanbeimu (Fritillaria cirrhosa D.Don) 6g; Banxia (Pinellia ternata (Thunb.) Breit.) 10g.
Zhang 2011 (31)	Qinlian Pingwei Decoction	Huangqin (Scutellaria baicalensis Georgi) 10g; Cangzhu (Atractylodes lancea (Thunb.) DC.) 10g; Houpo (Magnolia officinalis Rehd. et Wils.) 10g; Zhuling (Wolfiporia cocos (F.A. Wolf) Ryvarden & Gilb.) 10g; Chenpi (Citri Reticulatae Pericarpium) 6g; Huanglian (Coptis chinensis Franch.) 6g; Gancao (Glycyrrhiza uralensis Fisch.) 3g.
Zhang 2015 (32)	Jianpi Qingre Lishi Decoction	Danshen (Salvia miltiorrhiza Bunge) 20g; Shichangpu (Acorus tatarinowii Schott) 20g; Zelan (Eupatorium fortunei Turcz.) 15g; Zhuling (Wolfiporia cocos (F.A. Wolf) Ryvarden & Gilb.) 15g; Caodoukou (Amomum kravanh Pierre ex Gagnep.) 15g; Biandou (Vigna umbellata (Thunb.) Ohwi et Ohashi) 15g; Huanglian (Coptis chinensis Franch.) 10g; Huangqin (Scutellaria baicalensis Georgi) 10g; Jianghuang (Curcuma aromatica Salisb.) 10g; Zhiqiao (Fructus Aurantii Immaturus) 10g; Zhuru (Caulis Bambusae In Taeniam) 10g; Chenpi (Citri Reticulatae Pericarpium) 6g.
Zhang 2016 (33)	Qingre Lishi Decoction	Huanglian (Coptis chinensis Franch.) 9g; Sangbaipi (Morus alba L. Cortex) 10g; Dihuang (Rehmannia glutinosa (Gaertn.) Libosch.) 20g; Tianhuafen (Trichosanthes kirilowii Maxim. Radix) 20g; Yiyiren (Coicis Semen) 15g; Zelan (Eupatorium fortunei Turcz.) 10g; Danshen (Salvia miltiorrhiza Bunge) 15g; Gegen (Pueraria lobata (Willd.) Ohwi Radix) 20g; Shanyao (Dioscorea oppositifolia L. Rhizoma) 15g.
Zhang 2023 (34)	Jianpi Qingre Lishi Decoction+Metformin	Gegen (Pueraria lobata (Willd.) Ohwi Radix) 30g; Shanzha (Crataegus pinnatifida Bge.) 30g; Shanyao (Dioscorea oppositifolia L. Rhizoma) 30g; Gualou (Trichosanthes kirilowii Maxim.) 15g; Baizhu (Atractylodes macrocephala Koidz.) 15g; Chenpi (Citrus reticulata Blanco Pericarpium) 12g; Huanglian (Coptis chinensis Franch.) 9g; Banxia (Pinellia ternata (Thunb.) Breit.) 9g; Huangqin (Scutellaria baicalensis Georgi) 9g; Gancao (Glycyrrhiza uralensis Fisch.) 6g.
Zhi 2024 (35)	Jianpi Qingre Lishi Decoction+Metformin, GLP-1	Shanyao (Dioscorea oppositifolia L. Rhizoma) 30g; Shanzha (Crataegus pinnatifida Bge.) 30g; Gegen (Pueraria lobata (Willd.) Ohwi Radix) 30g; Baizhu (Atractylodes macrocephala Koidz.) 15g; Gualoushi (Trichosanthes kirilowii Maxim. Fructus) 15g; Chenpi (Citrus reticulata Blanco Pericarpium) 12g; Huangqin (Scutellaria baicalensis Georgi) 9g; Banxia (Pinellia ternata (Thunb.) Breit.) 9g; Huanglian (Coptis chinensis Franch.) 9g; Gancao (Glycyrrhiza uralensis Fisch.) 6g.

### 3.3 Bias risk assessment results

Among the 18 included studies, 17 were RCTs, and one was a cohort study. The quality of the RCTs was evaluated by the Cochrane RoB tool. In terms of random sequence generation, the eligible studies were identified as low risk, except for two studies (20, 24) that did not report the randomization process. Regarding allocation concealment, one study (20) was assessed as unclear risk as it did not specify the method. All studies employed blinding and reported treatment efficacy. Regarding the integrity of outcome data, one study (22) was rated as having an unclear risk as it did not provide sufficient details. In terms of selection bias, one study (20) was assessed as high risk as it specifically targeted an older population. Regarding other biases, all studies were assessed as low risk. The NOS was utilized to assess the quality of the cohort study. Except for the unclear risk of the comparability for additional factors, the cohort study was assessed as low risk for the other items, with an overall score of eight.

### 3.4 Change in FBG

**Figure 2:**
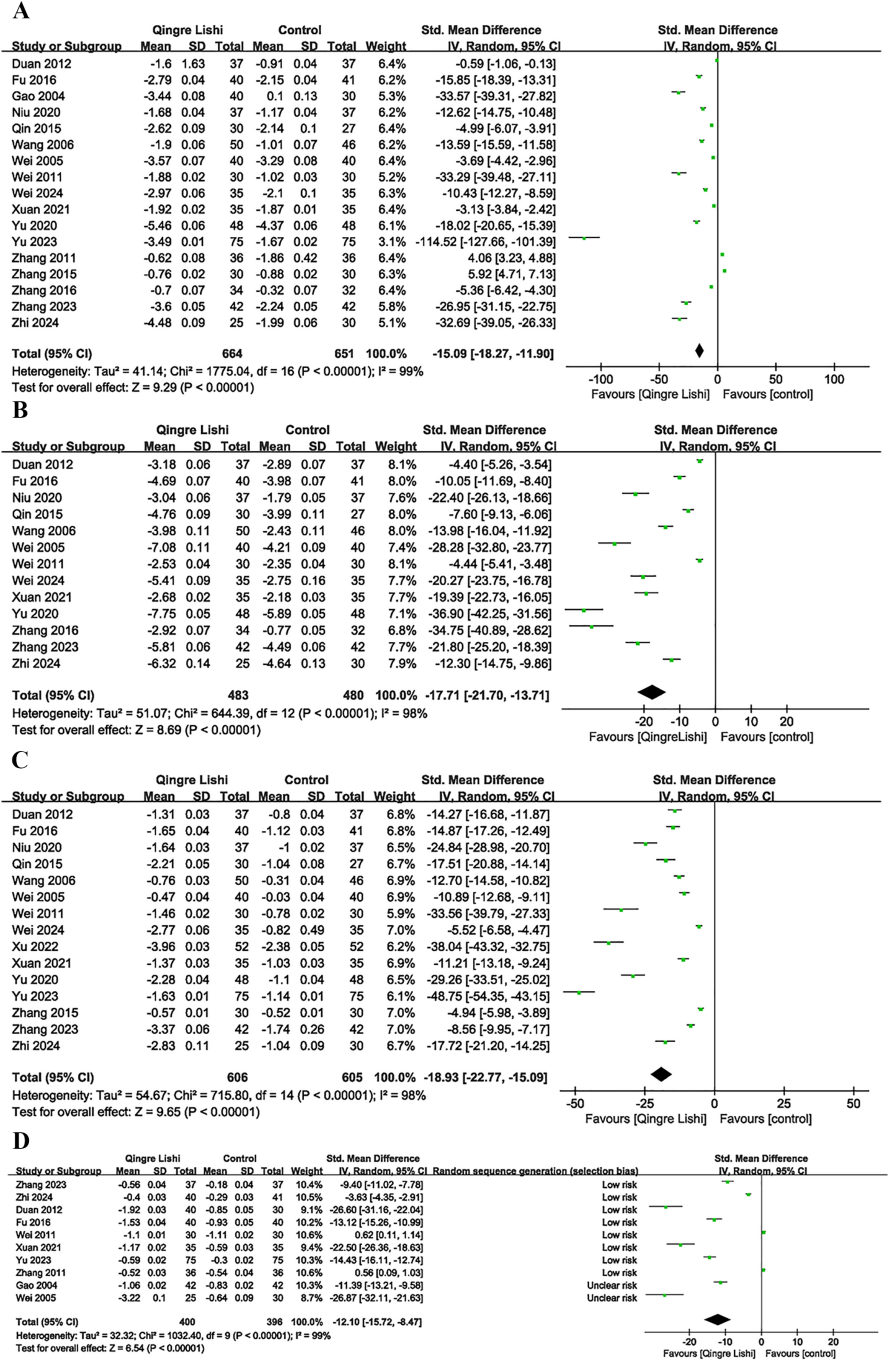
Forest plot. **(A)** FBG; **(B)** 2hPG; **(C)** HbA1c; **(D)** TC.

Among the 18 studies, 17 measured the levels of FBG as an outcome indicator. The results demonstrated that QRLSD, as an adjunct therapy, was significantly more effective in lowering FBG levels than treatments used in the control group (**Figure 2A**; SMD: -15.09; 95% CI: [-18.27, -11.90], I² = 99%, P < 0.00001). Subgroup analyses based on the changes in FBG were carried out. Seven subgroups were stratified by different interventions combined with QRLSD. The results showed that the efficacy of QRLSD and interventions combined with QRLSD was superior to that of the treatments employed in the control group. Out of the eligible studies, four employed QRLSD as a standalone intervention, seven combined it with metformin, one combined it with diamicron MR, one combined it with both diamicron MR and metformin, one combined it with dapagliflozin, and one combined it with metformin and GLP-1 agonists. The results all demonstrated statistical significance. Two subgroups were stratified by intervention duration (12 weeks and above vs. less than 12 weeks), and the results indicated that QRLSD was effective in lowering FBG levels in both subgroups. Two subgroups were stratified by patient age (55 years and above vs. below 55 years), and the results demonstrated that QRLSD effectively reduced FBG levels in both subgroups, with P values indicating statistical significance. The subgroup analyses are illustrated in **Table 3**.

**Table 3:** Subgroup analysis.

Subgroup	Change in FBG				Change in 2hpG				Change in HbA1c			
	Study	SMD [95%CI]	*P* value	*I* ^2^	Study	SMD [95%CI]	*P* value	*I* ^2^	Study	SMD [95%CI]	*P* value	*I* ^2^
Total	17	-15.09 [-18.27, -11.90]	<0.00001	0.99	13	-17.71 [-21.7, -13.71]	<0.00001	0.98	15	-18.93 [-22.77, -15.09]	<0.00001	0.98
Intervention												
Qingre Lishi alone	4	-1.23 [-5.38, -2.91]	<0.00001	0.99	2	-27.28 [-41.47, -13.09]	<0.00001	0.94	3	-8.26 [-12.38, -4.13]	<0.00001	0.97
Qingre Lishi +insulin	——	——	——	——	——	——	——	——	1	-38.04 [-43.32, -32.75]	<0.00001	NA
Qingre Lishi +Metformin	7	-11.43 [-15.15, -7.72]	<0.00001	0.98	8	-19.73 [-25.14, -14.32]	<0.00001	0.97	8	-11.37 [-15.54, -2.21]	<0.00001	0.99
Qingre Lishi n+Diamicron	1	-33.57 [-39.31, -27.82]	<0.00001	NA	——	——	——	——	1	——	——	——
Qingre Lishi +Diamicron,Metformin	1	-33.44 [-37.65, -29.23]	<0.00001	NA	1	-4.44 [-5.41, -3.48]	<0.00001	NA	1	-33.56 [-39.79, -27.33]	<0.00001	NA
Qingre Lishi +Dapagliflozin	1	-114.52 [-127.66, -101.39]	<0.00001	NA	——	——	——	——	1	-48.75 [-54.35, -43.15]	<0.00001	NA
ianpi Qingre Lishi +Metformin,GLP-1	1	-32.69 [-32.05, -26.33]	<0.00001	NA	1	-12.3 [-14.75, -9.86]	<0.00001	NA	1	-17.72 [-21.2, -14.25]	<0.00001	NA
Treatment time												
≥12 weeks	8	-23.43 [-31.16, -15.69]	<0.00001	0.99	6	-18.39 [-26.17, -10.6]	<0.00001	0.99	8	-19.72 [-25.81, -13.62]	<0.00001	0.98
<12 weeks	9	-10.56 [-14.19, -6.94]	<0.00001	0.99	7	-17.3 [-22.92, -11.69]	<0.00001	0.98	7	-18.2 [-23.88, -12.51]	<0.00001	0.98
Mean/median age												
≥55y	6	-18.71 [-24.55, -12.88]	<0.00001	0.99	4	-11.39 [-16.83, -5.95]	<0.00001	0.97	6	-26.63 [-35.63, -17.62]	<0.00001	0.97
<55y	9	-11.37 [-15.54, -7.21]	<0.00001	0.99	8	-22.27 [-30.26, -14.27]	<0.00001	0.99	8	-13.62 [-17.24, 10.01]	<0.00001	0.97

### 3.5 Change in 2hpG

A total of 13 studies included 2hPG as an outcome indicator (**Figure 2B**; SMD: -17.71; 95% CI: [-21.7, -13.71], I² = 98%, P < 0.00001). The results indicated that QRLSD, as an adjunct therapy, effectively reduced 2hPG levels and outperformed the treatments used in the control group. Subgroup analyses based on the changes in 2hPG were carried out. Seven subgroups were stratified by different interventions combined with QRLSD. The results demonstrated that the QRLSD and interventions combined with QRLSD both effectively lowered the 2hPG levels. Among the included studies, two employed the QRLSD alone, eight combined it with metformin, one combined it with metformin and diamicron MR, and one combined it with metformin and GLP-1 agonists, with all results suggesting p < 0.00001. Two subgroups were stratified by intervention duration (12 weeks and above vs. less than 12 weeks), and the results demonstrated that QRLSD was more effective in lowering 2hPG levels than treatments used in the control group. Two subgroups were stratified by patient age (55 years and above vs. below 55 years), and the results showed that QRLSD effectively reduced 2hPG levels in both subgroups.

### 3.6 Change in HbA1c

A total of 15 studies included HbA1c as an outcome indicator (**Figure 2C**; SMD: -18.93; 95% CI: [-22.77, -15.09], I² = 98%, P < 0.00001). The results demonstrated that QRLSD reduced the levels of HbA1c, an indicator of blood glucose changes over time, as an adjunct therapy for treating T2D and was more effective than the treatments used in the control group. Subgroup analyses based on the changes in HbA1c were carried out. Seven subgroups were stratified by different interventions combined with QRLSD. The results showed that the QRLSD and interventions combined with QRLSD both effectively lowered the HbA1c levels. Among the included studies, three used QRLSD alone, eight combined it with metformin, one combined it with insulin, one combined it with metformin and diamicron MR, one combined it with dapagliflozin, and one combined it with metformin and GLP-1 agonists. The results all demonstrated the efficacy of these interventions in reducing HbA1c levels, with P values all suggesting statistical significance. Two subgroups were stratified by intervention duration (12 weeks and above vs. less than 12 weeks), and the results were statistically significant in both subgroups. Two subgroups were stratified by patient age (55 years and above vs. below 55 years), and the results indicated that QRLSD was effective in lowering HbA1c levels in both subgroups.

### 3.7 Change in blood lipids

**Figure 3:**
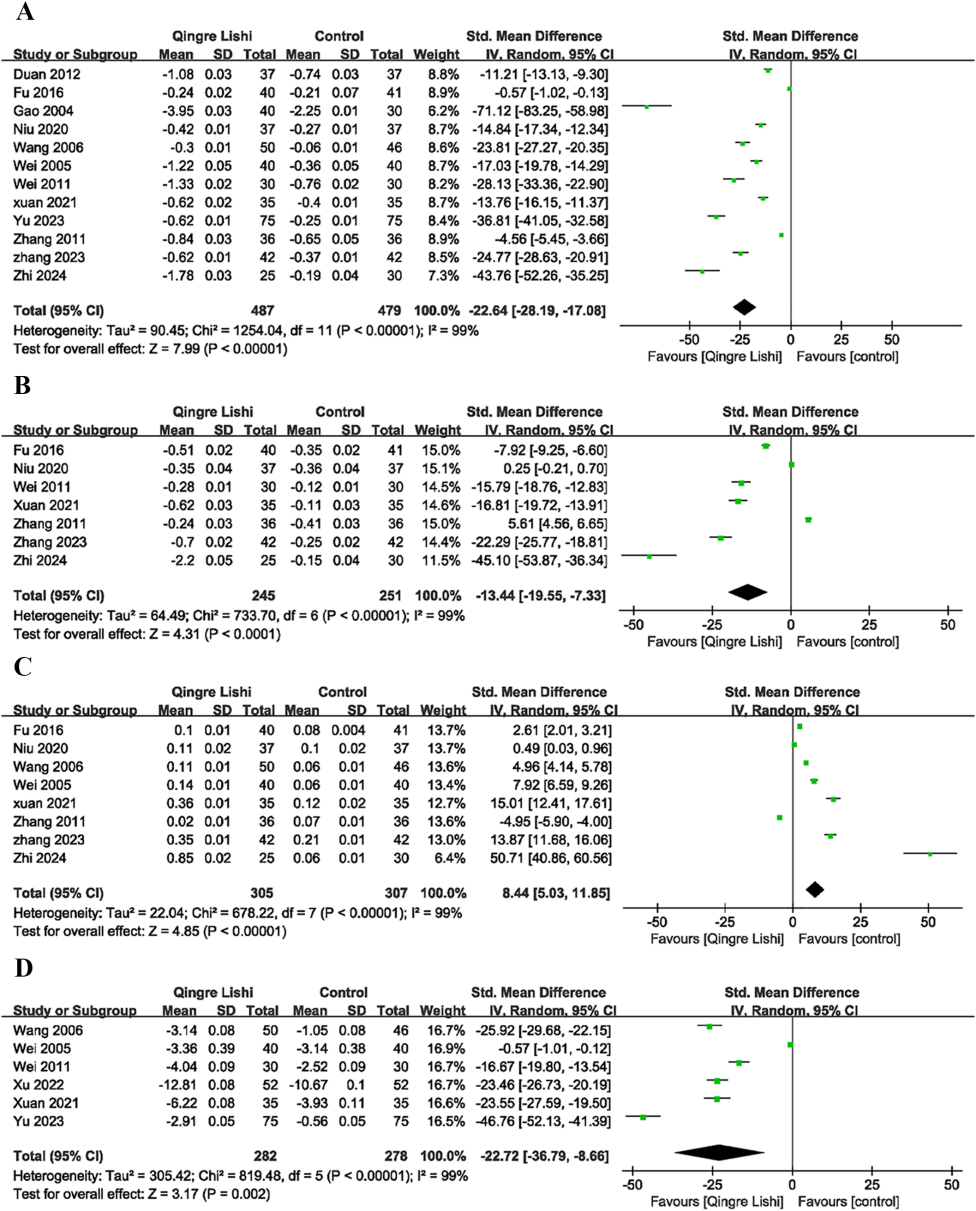
Forest plot. **(A)** TG; **(B)** LDL-C; **(C)** HDL-C; **(D)** FINs.

Some studies included the efficacy of QRLSD in modulating lipid metabolism as the outcome indicator, and reported its positive impact on blood lipid. The results demonstrated that QRLSD, as an adjunct therapy, effectively improved lipid metabolism. The evaluation indicators included improvements in total cholesterol (TC) (**Figure 2D**; SMD: -12.1; 95% CI: [-15.72, -8.47], I² = 99%, P < 0.00001), triglycerides (TG) (**Figure 3A**; SMD: -22.64; 95% CI: [-28.19, -17.08], I² = 99%, P < 0.00001), low-density lipoprotein cholesterol (LDL-C) (**Figure 3B**; SMD: -13.44; 95% CI: [-19.55, -7.33], I² = 99%, P < 0.00001), and high-density lipoprotein cholesterol (HDL-C) (**Figure 3C**; SMD: 8.44; 95% CI: [5.03, 11.85], I² = 99%, P <0.00001).

### 3.8 Change in insulin assessment parameters

**Figure 4:**
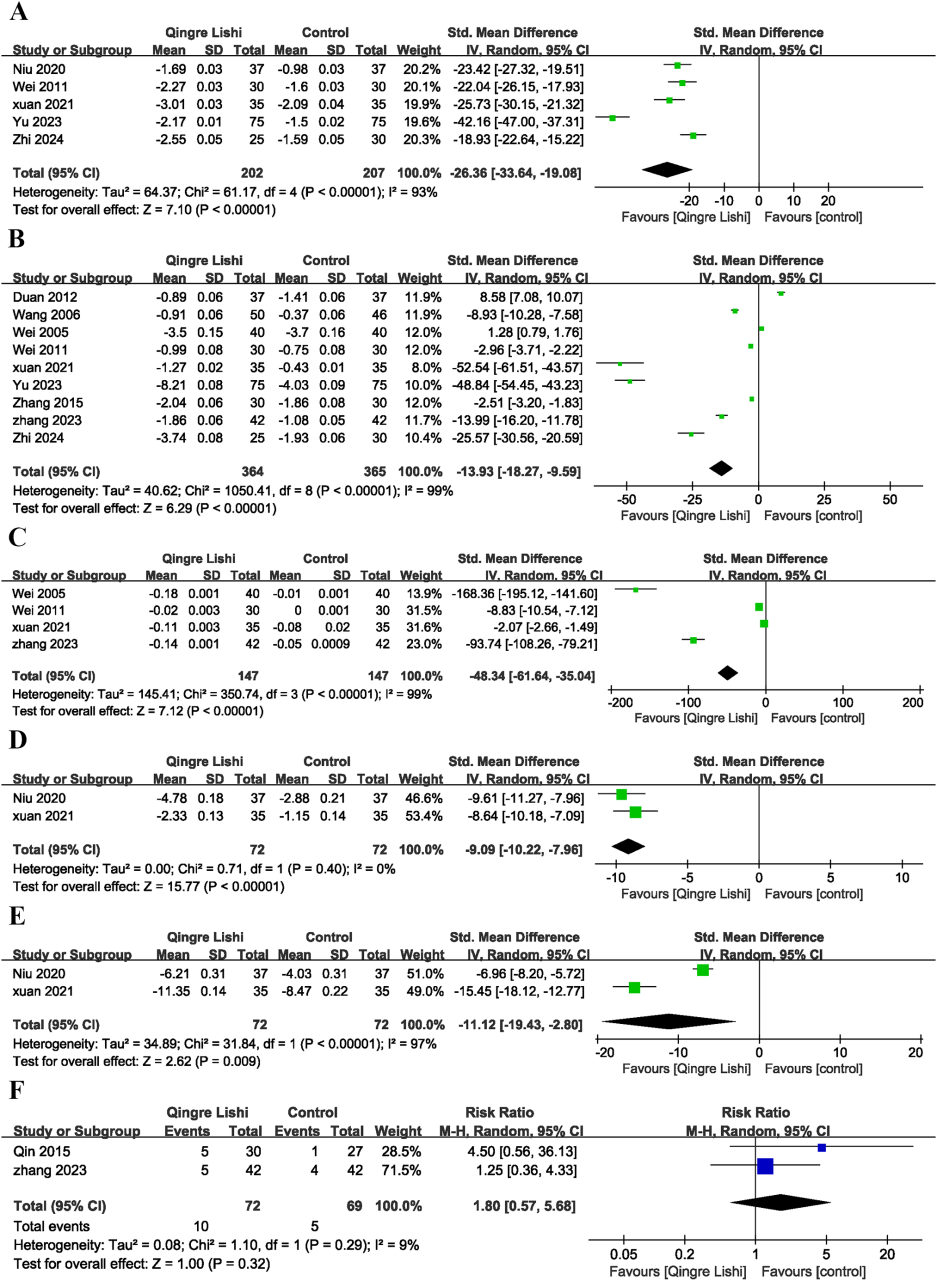
Forest plot. **(A)** HOME-IR; **(B)** BMI; **(C)** WHR; **(D)** Weight; **(E)** Waistline; **(F)** Plot of adverse effects.

Considering the impact of T2D on insulin sensitivity, several studies also measured insulin-related indicators. Six studies measured the levels of fasting insulin (FINs) (**Figure 3D**; SMD: -22.72; 95% CI: [-36.79, -8.66], I² = 99%, P=0.002), and five measured the homeostasis model assessment for insulin resistance (HOMA-IR) index (**Figure 4A**; SMD: -26.36; 95% CI: [-33.64, -19.08], I² = 93%, P=0.01). The results showed that QRLSD improved insulin-related indicators as an adjuvant therapy.

### 3.9 Change in anthropometric indicators

Several studies reported changes in BMI (**Figure 4B**; SMD: -13.93; 95% CI: [-18.27, -9.59], I² = 99%, P < 0.00001), waist-to-hip ratio (WHR) (**Figure 4C**; SMD: -48.34; 95% CI: [-61.64, -35.04], I² = 99%, P <0.00001), weight (**Figure 4D**; SMD: -9.09; 95% CI: [10.22, -7.96], I² = 0%, P <0.00001), and waist circumference (**Figure 4E**; SMD: -11.12; 95% CI: [-19.43, -4.15], I² = 97%, P=0.009). The results demonstrated that QRLSD, as an adjuvant therapy, was effective in treating T2D, suggesting its role in improving anthropometric indicators.

### 3.10 Adverse effects

Adverse effects analysis suggested that T2D patients receiving QRLSD as the adjuvant treatment had no significant adverse effects, and the results demonstrated no statistical significance (**Figure 4F**; RR: 1.8; 95% CI: [0.57, 5.68], I² = 9%, P=0.32).

### 3.11 Publication bias and sensitivity analysis

**Figure 5:**
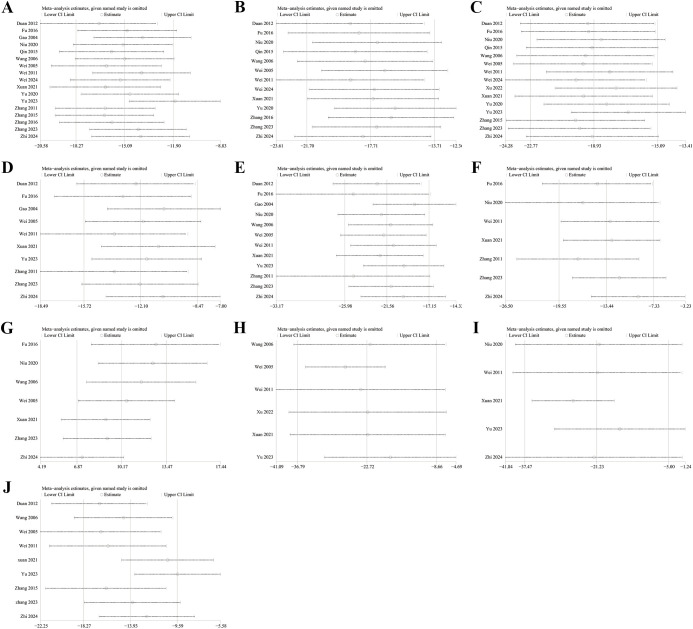
Sensitivity analysis. **(A)** FBG; **(B)** 2hPG; **(C)** HbA1c; **(D)** TC; **(E)** TG; **(F)** LDL-C; **(G)** HDL-C; **(H)** FINs; **(I)** HOME-IR; **(J)** BMI.

**Figure 6:**
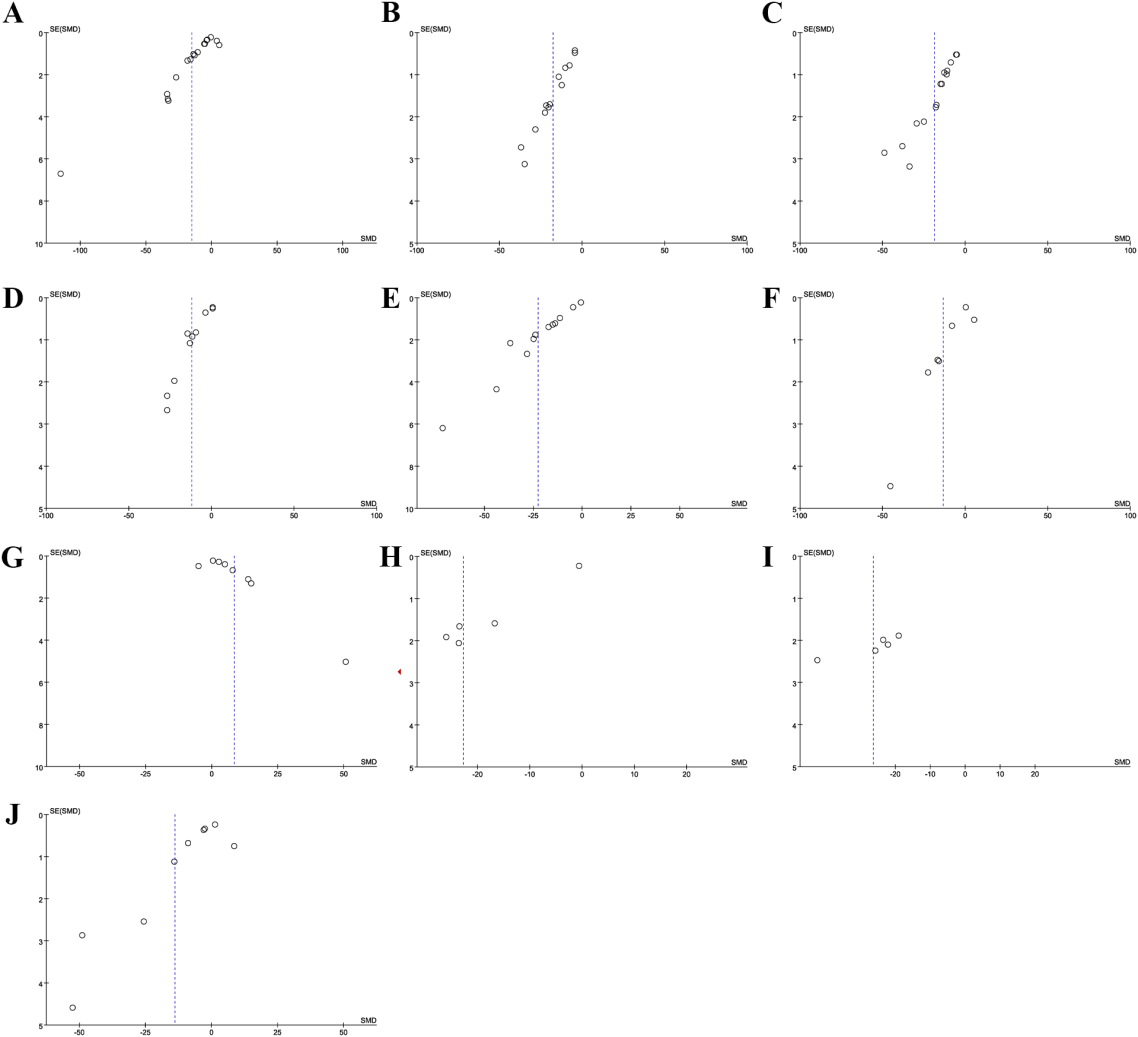
Funnel plot. **(A)** FBG; **(B)** 2hPG; **(C)** HbA1c; **(D)** TC; **(E)** TG; **(F)** LDL-C; **(G)** HDL-C; **(H)** FINs; **(I)** HOME-IR; **(J)** BMI.

Sensitivity analyses for FBG (**Figure 5A**), 2hpG (**Figure 5B**), HbA1c (**Figure 5C**), TC (**Figure 5D**), TG (**Figure 5E**), LDL-C (**Figure 5F**), HDL-C (**Figure 5G**), FINs (**Figure 5H**), HOMA-IR (**Figure 5I**), and BMI (**Figure 5J**) demonstrated that the results across all indicators were stable. Funnel plots and Egger’s tests demonstrated significant publication bias in FBG (P=0.0001; **Figure 6A**), 2hPG (P=0.0001; **Figure 6B**), HbA1c (P=0.0001; **Figure 6C**), TC (P=0.0001; **Figure 6D**), TG (P=0.0001; **Figure 6E**), LDL-C (P=0.039; **Figure 6F**), HDL-C (P=0.0001; **Figure 6G**), FINs (P=0.0001; **Figure 6H**), OME-IR(P=0.004; **Figure 6I**), and BMI (P=0.027; **Figure 6J**).

### 3.12 GRADE assessment

The GRADE approach was used to assess the quality of evidence for all outcomes (**Table 4**). A total of 14 outcomes were evaluated. For glycemic outcomes, including changes in FBG, 2hPG, and HbA1c, the evidence was rated as high. However, inconsistency was considered serious, primarily due to heterogeneity among patient populations, such as differences in age and BMI. Regarding other considerations, publication bias was detected based on funnel plots and Egger’s test, which was accounted for as a reporting bias.

**Table 4:** GRADE assessment.

Outcome Measures	Design	Risk of bias	Inconsistency	Indirectness	Imprecision	Other considerations	Quality
Change in FBG	Randomized trials	No serious risk of bias	Serious	No serious indirectness	No serious imprecision	Reporting bias very strong association	HIGH
Change in 2hPG	Randomized trials	No serious risk of bias	Serious	No serious indirectness	No serious imprecision	Reporting bias very strong association	HIGH
Change in HbA1c	Randomized trials	No serious risk of bias	Serious	No serious indirectness	No serious imprecision	Reporting bias very strong association	HIGH
Change in TC	Randomized trials	No serious risk of bias	Serious	No serious indirectness	No serious imprecision	Reporting bias very strong association	MODERATE
Change in TG	Randomized trials	No serious risk of bias	Serious	No serious indirectness	No serious imprecision	Reporting bias very strong association	HIGH
Change in BMI	Randomized trials	No serious risk of bias	serious	No serious indirectness	No serious imprecision	very strong association	HIGH
Change in LDL-C	Randomized trials	No serious risk of bias	Serious	No serious indirectness	No serious imprecision	Reporting bias very strong association	HIGH
Change in HDL-C	Randomized trials	No serious risk of bias	Serious	No serious indirectness	No serious imprecision	Reporting bias very strong association	MODERATE
Change in HOM-IR	Randomized trials	No serious risk of bias	Serious	No serious indirectness	No serious imprecision	Reporting bias very strong association	MODERATE
Change in FINs	Randomized trials	No serious risk of bias	Serious	No serious indirectness	No serious imprecision	Reporting bias very strong association	HIGH
Change in weight	Randomized trials	No serious risk of bias	Serious	No serious indirectness	Very serious	Strong association	MODERATE
Change in waistline	Randomized trials	No serious risk of bias	No serious inconsistency	No serious indirectness	Very serious	Very strong association	HIGH
Change in WHR	Randomized trials	No serious risk of bias	Serious	No serious indirectness	No serious imprecision	Very strong association	HIGH
Change in adverse effects	Randomized trials	No serious risk of bias	No serious inconsistency	No serious indirectness	Very serious	None	HIGH

For lipid-related outcomes, the quality of evidence for improvements in TG and LDL-C was rated as high, and risk factors were similarly stemmed from population differences and potential risk of bias. The quality of evidence for changes in TC and HDL-C was rated as moderate. Although the evidence was directly applicable to the outcomes and sensitivity analyses indicated stability, heterogeneity among populations and risk of bias persisted. For insulin-related outcomes, the quality of evidence for change in FINs was rated as high, and the six included studies differed in population characteristics and were subject to publication bias. The quality of evidence for HOMA-IR improvement was rated as moderate, and the five included studies varied in population characteristics and intervention methods, and were also subject to publication bias. For anthropometric outcomes, the quality of evidence for BMI improvement was rated as high. Although there were differences in baseline body weight and intervention methods, no publication bias was detected. The quality of evidence for waist circumference and WHR was also rated as high, with no publication bias. Waist circumference showed no serious inconsistency. However, due to the small sample size, the precision was rated as very serious. The quality of evidence for weight was rated as moderate, with risks of inconsistency and imprecision. The quality of evidence for adverse events was rated as high. Although the number of included participants was small, the results exhibited a clearly defined null effect range.

## 4 Discussion

China currently has the largest number of people with diabetes globally, accounting for approximately one-quarter of the world’s total cases. Since the 1970s, lifestyle changes, stress, obesity, and aging have contributed to a persistently high prevalence of T2D in China (36). By 2050, the global death toll from diabetes is projected to reach 1.59 million. While the death rate of type 1 diabetes in China has declined, T2D mortality remains uncontrolled (37). Early diagnosis and treatment of T2D are crucial for improving patients’ quality of life, reducing death rates, and alleviating financial burdens. The treatment of T2D has long been researched in TCM, and it is defined as an emaciation-thirst disease. The classic symptoms of this disease are “three excesses and one deficiency” (polyuria, polydipsia, polyphagia, and weight loss). TCM attributes the pathogenesis of T2D to “stagnation, heat, deficiency, and damage,” corresponding to the four stages of diabetes. The treatment of T2D primarily focuses on “heat and deficiency” (38). Some researchers have proposed the “damp-heat-induced thirst” theory, suggesting that damp-heat is the dominant syndrome in T2D patients. Moreover, they have conducted clinical studies based on QRLSD (39).

Our results showed that QRLSD significantly reduced the levels of FBG, 2hPG, and HbA1c. Additionally, QRLSD lowered the values of TC, LDL-C, and TG, as well as elevated the values of HDL-C, suggesting its role in improving metabolism. Furthermore, QRLSD decreased the values of HOMA-IR and FINs, and lowered BMI, WHR, and the values of WC and body weight. Sensitivity analyses indicated stable outcomes across the included studies. Egger’s test suggested potential publication bias, which is a crucial factor affecting the quality of the study.

In recent years, studies focused on TCM decoctions have increased due to their efficacy and minimal side effects. Studies on TCM formulations for treating diabetes have continued, including both classical prescriptions and new therapeutic approaches developed by modern TCM practitioners. A review by X Meng (40) demonstrated that TCM decoctions can reduce insulin resistance, increase the expression of insulin receptors, and stimulate insulin secretion from pancreatic β cells, thus effectively controlling blood glucose and mitigating target organ damage. Numerous systematic reviews have also examined the efficacy of TCM decoctions in treating T2D. B Zhou (41) conducted a systematic review on the efficacy of Xiaoke decoction in treating T2D. The results showed that conventional Western medicine treatment combined with Xiaoke decoction was more effective than single Western medicine therapy in improving overall efficacy and lowering the levels of FBG, 2hPG, and HbA1c. Z Zhang (42) evaluated the efficacy of Da Chaihu decoction (DCHD) in treating T2D through a systematic review. It is suggested that when used alone, DCHD can reduce the levels of blood glucose and is relatively safe. Moreover, Western medicine therapy combined with DCHD can provide superior efficacy in regulating glucose-lipid metabolism and improving pancreatic function. A systematic review by Y Tian (43) demonstrated that Huanglian Wendan decoction significantly lowered the levels of blood glucose and lipids when used as an adjunct therapy. These findings validate the reliability of integrating TCM decoctions into T2D treatment.

T2D is closely linked to inflammatory cytokines, with elevated serum levels of tumor necrosis factor-alpha and interleukin-6 observed in T2D patients. These inflammatory cytokines can impair signal transduction by interfering with insulin-binding sites in insulin signaling pathways, thus causing insulin resistance (44). Additionally, recent studies suggested a strong correlation between gut microbiota and T2D. It indicated that microbiota dysbiosis can trigger inflammation, thereby causing impaired intestinal permeability and glucose-lipid metabolism (45). In TCM, inflammatory diseases are often defined as damp-heat syndrome, and QRLSD can modulate inflammation levels (10).

TCM decoctions are known for their multi-target, holistic regulatory effects. The glucose-lowering pharmacological effects of the most frequently used herbs are summarized in **Table 5** (46–64). For compound formulations, these advantages are even more pronounced. GQD, a QRLSD formula, was commonly used in treating T2D. A study indicated that GQD can modulate gut microbiota and maintain intestinal homeostasis (65). Berberine, one of its key components, plays a critical role in modulating gut microbiota for treating T2D and reducing systemic and localized inflammation. Moreover, GQD can prevent high-fat diet-induced obesity and reduce the body weight of the mice models. Compared to metformin, berberine exhibits superior efficacy in regulating gut microbiota (66). Other commonly used QRLSD formulations include YinChen WuLing Powder and YCHD. Qinghao (artemisia annua), a primary component, can regulate metabolism by downregulating the expression of fatty acid synthase via the miR-122 pathway, thereby reducing the levels of blood glucose and lipid (67). A study demonstrated that YCHD can modulate inflammation, remodel vessels, and inhibit the formation and metabolism of adipose tissue. Moreover, it showed that compared to single herbs, decoction formula can significantly enhance the bioactivity of some active components (68). A study indicated that, in comparison to the control group receiving insulin, the levels of interleukin-1 beta, monocyte chemoattractant protein-1, and HMGB1 significantly decreased in T2D patients receiving interventions combined with QRLSD. It suggested that active components such as coptis chinensis and anemarrhena asphodeloides contributed to anti-inflammatory effects (27).

**Table 5:** Pharmacological actions of high-frequency drugs for glucose lowering.

Serial number	Name	Number of times	Frequency (n=175)	Hypoglycemic pharmacological effects
1	Huanglian	14	77.78%	Improving insulin resistance, promoting glycolysis, regulating the function of liver and pancreatic β-cells, and modulating blood lipids and gut microbiota (46).
2	Gancao	11	61.11%	Improving insulin sensitivity, enhancing glucose tolerance, regulating glucose and lipid metabolism, and increasing insulin secretion (47).
3	Shanyao	10	55.56%	Improving insulin resistance, inhibiting α-glucosidase activity, delaying intestinal glucose absorption, suppressing DPP-IV activity, increasing endogenous GLP-1 concentration, exerting antioxidant effects, and regulating immune function (48).
4	Fuling	8	44.44%	Lowering blood glucose, blood lipids, and insulin resistance, reducing inflammatory response, and protecting the intestinal barrier (49).
5	Huangqin	7	38.89%	Activating α-glucosidase activity, promoting glycogen synthesis, alleviating insulin resistance, and enhancing the body’s sensitivity to insulin as well as glucose transport capacity (50).
6	Cangzhu	7	38.89%	Improving insulin resistance, modulating the structure and metabolism of gut microbiota, and exerting antioxidant and anti-glycation effects (51).
7	Banxia	7	38.89%	Exerting antioxidant effects, reducing lipid levels, improving insulin sensitivity index, and promoting glycogen synthesis (52).
8	Chenpi	6	33.33%	Inhibiting adipogenesis, regulating genes associated with lipogenesis, modulating the gut microbiota, and influencing pathways of lipid and cholesterol homeostasis (53).
9	Gegen	5	27.78%	Upregulating the expression of the glucagon-like peptide-1 receptor (GLP-1R), promoting the neogenesis of pancreatic β-cells, improving insulin resistance, and lowering blood glucose levels (54).
10	Danshen	5	27.78%	Enhancing insulin sensitivity, boosting mitochondrial activity, and suppressing associated inflammation and oxidative stress (55).
11	Huangqi	4	22.22%	Lowering blood glucose, exerting anti-inflammatory effects, protecting the intestinal barrier, and improving vascular endothelial dysfunction (56).
12	Baizhu	4	22.22%	Enhancing insulin sensitivity and improving glucose and lipid metabolism (57).
13	Dihuang	4	22.22%	Inhibiting hepatic gluconeogenesis and increasing hepatic glycogen synthesis, elevating serum insulin levels, and suppressing hepatic steatosis (58).
14	Zexie	4	22.22%	Inhibiting hepatic lipid accumulation and steatosis, protecting pancreatic islet cells, suppressing inflammation, exerting antioxidant effects, and inhibiting α-glucosidase activity (59).
15	Shanzha	4	22.22%	Countering insulin resistance, improving lipid metabolism, promoting glucose uptake, and reducing hyperglycemia-induced inflammatory responses and apoptosis in cells (60).
16	Yinchen	4	22.22%	Exerting anti-inflammatory effects and reducing hepatic lipid accumulation (61).
17	Dangshen	3	16.67%	Improving glucose metabolism, alleviating insulin resistance, and ameliorating lipid metabolic disorders (62).
18	Yiyiren	3	16.67%	Protecting and repairing pancreatic β-cells, regulating inflammatory factors, modulating lipid and protein metabolism, inhibiting intestinal glucose absorption, promoting glucose utilization, ameliorating insulin resistance through gene regulation, and modulating gut microbiota (63).
19	Shengjiang	3	16.67%	Promoting glucose uptake, stimulating the expression of glucose transporter 4 (GLUT4), inhibiting protein glycation, and elevating serum insulin levels (64).

A total of 18 studies assessing the efficacy of QRLSD for T2D were included. The efficacy and safety of QRLSD in treating T2D were validated through a comprehensive analysis. Moreover, heterogeneity among major outcomes was investigated through subgroup analyses. Therefore, our findings may provide evidence-based support for further clinical studies.

Due to substantial heterogeneity in some key outcomes, sensitivity analyses were conducted and suggested stable results. To explore potential sources of this heterogeneity, data from the included studies were examined. Notably, the composition of QRLSD varied. The formulations were designed to achieve the effect of clearing heat and removing dampness, and drew on both historical knowledge and clinical experience. The selected herbs belonged to the same functional category but differed in type, which may contribute to heterogeneity. Additionally, intervention methods varied, with some studies applying QRLSD alone and others combining it with other therapies, further contributing to heterogeneity. Subgroup analyses indicated that, for key indicators including FBG, HbA1c, and 2hPG, both QRLSD alone and in combination with Western medicine were effective. The included RCTs and cohort studies involved populations of different ages and baseline indicators. In the intervention groups, mean ages ranged from 44.06 ± 9.55 to 67.27 ± 4.44 years, and in the control groups, from 45.94 ± 10.38 to 65.89 ± 3.84 years. Subgroup analysis using 55 years as a cutoff showed that QRLSD improved key glycemic indicators regardless of age. Follow-up durations also varied across studies, ranging from 4 to 24 weeks, which could contribute to heterogeneity. Subgroup analysis using 12 weeks as a cutoff revealed that the treatment was effective regardless of whether the follow-up duration was longer or shorter than 12 weeks. The included literature comprised 17 RCTs and one cohort study, and differences in study design may also account for some heterogeneity.

Some RCTs did not report allocation concealment or blinding, which may introduce a non-negligible risk of bias. The sample size meeting the inclusion criteria was relatively small, and most studies originated from China. Therefore, significant regional variations within China, along with differences in individual constitutions and local backgrounds, may contribute to publication bias and region-specific selection bias. In addition, studies with positive outcomes are more likely to be published, which could be a source of publication bias. Therefore, further high-quality trials are needed to strengthen the level of evidence.

## 5 Conclusion

QRLSD can improve the management of T2D by lowering the levels of FBG, 2hPG, HbA1c, LDL-C, BMI, TC, TG, HDL-C, HOMA-IR, body weight, WC, and WHR. Moreover, when combined with conventional Western medications, QRLSD demonstrated superior efficacy compared with Western medications alone, without causing significant adverse effects. These findings suggest that QRLSD may serve as a safe and effective comprehensive treatment option for T2D. However, as most of the included studies were conducted in China and potential heterogeneity and publication bias may exist, further international, multicenter, large-scale RCTs are required to validate the efficacy and safety of QRLSD in T2D management.

## Data Availability

The original contributions presented in the study are included in the article/**Supplementary Material**. Further inquiries can be directed to the corresponding author.

## Supplementary material

Supplementary File 1 Search strategy. PRISMA Checklist.
