# Supplementary material for: Efficacy and safety of Qingre Lishi decoction for type 2 diabetes: a systematic review and meta-analysis
Source: Front Endocrinol (Lausanne). 2025 Oct 20;16:1604633. doi: 10.3389/fendo.2025.1604633 (PMC12580096; doi:10.3389/fendo.2025.1604633)

**Supplementary File 1** Search strategy

Free word：

Adult-Onset Diabetes Mellitus

Ketosis-Resistant Diabetes Mellitus

Non-Insulin-Dependent Diabetes Mellitus

Stable Diabetes Mellitus

NIDDM

Maturity-Onset Diabetes Mellitus

Maturity Onset Diabetes Mellitus

MODY

Slow-Onset Diabetes Mellitus

Type 2 Diabetes Mellitus

Noninsulin-Dependent Diabetes Mellitus

Noninsulin Dependent Diabetes Mellitus

Maturity-Onset Diabetes

Maturity Onset Diabetes

Type 2 Diabetes

T2DM

Pubmed-1

((((((((((((((((Adult-Onset Diabetes Mellitus) OR (Ketosis-Resistant Diabetes Mellitus)) OR (Non-Insulin-Dependent Diabetes Mellitus)) OR (Stable Diabetes Mellitus)) OR (NIDDM)) OR (Maturity-Onset Diabetes Mellitus)) OR (Maturity Onset Diabetes Mellitus)) OR (MODY)) OR (Slow-Onset Diabetes Mellitus)) OR (Type 2 Diabetes Mellitus)) OR (Noninsulin-Dependent Diabetes Mellitus)) OR (Noninsulin Dependent Diabetes Mellitus)) OR (Maturity-Onset Diabetes)) OR (Maturity Onset Diabetes)) OR (Type 2 Diabetes)) OR (T2DM)) AND ((qingrelishi) OR (Qingre Lishi))

Web of science-1

((((((((((((((((Adult-Onset Diabetes Mellitus) OR (Ketosis-Resistant Diabetes Mellitus)) OR (Non-Insulin-Dependent Diabetes Mellitus)) OR (Stable Diabetes Mellitus)) OR (NIDDM)) OR (Maturity-Onset Diabetes Mellitus)) OR (Maturity Onset Diabetes Mellitus)) OR (MODY)) OR (Slow-Onset Diabetes Mellitus)) OR (Type 2 Diabetes Mellitus)) OR (Noninsulin-Dependent Diabetes Mellitus)) OR (Noninsulin Dependent Diabetes Mellitus)) OR (Maturity-Onset Diabetes)) OR (Maturity Onset Diabetes)) OR (Type 2 Diabetes)) OR (T2DM)) AND ((qingrelishi) OR (Qingre Lishi)) (Topic)

Embase-1


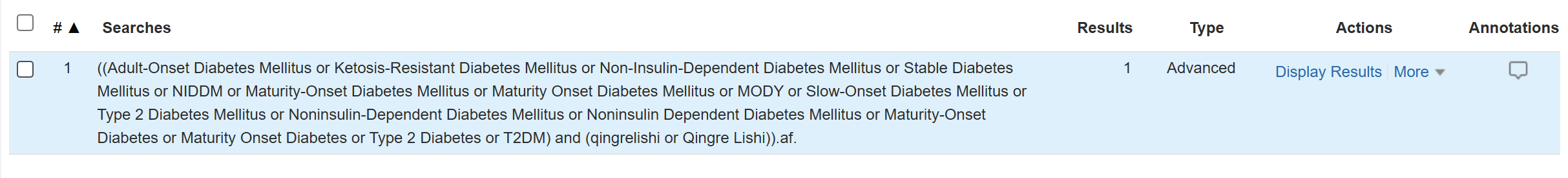


Cochrane-0


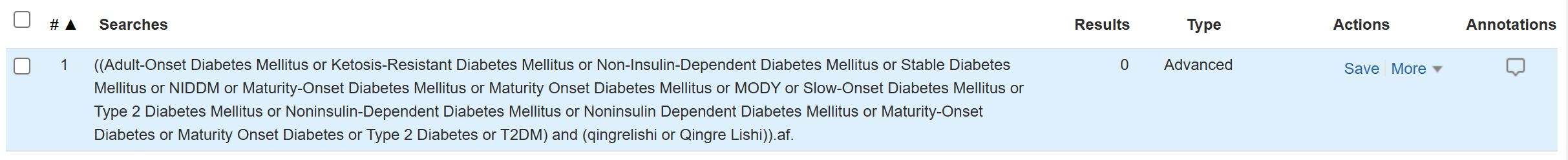


Wanfang-377


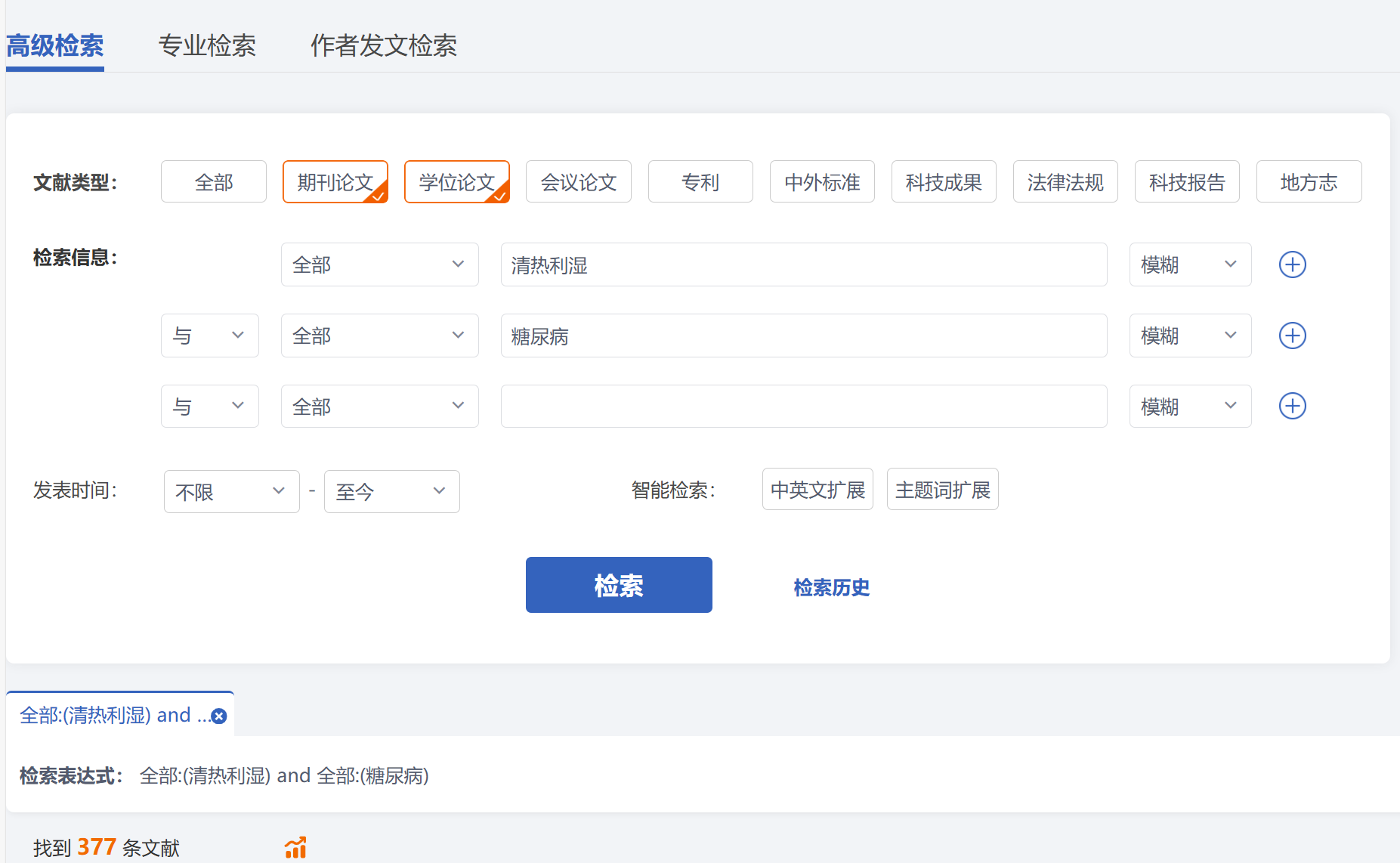


CnKi-326


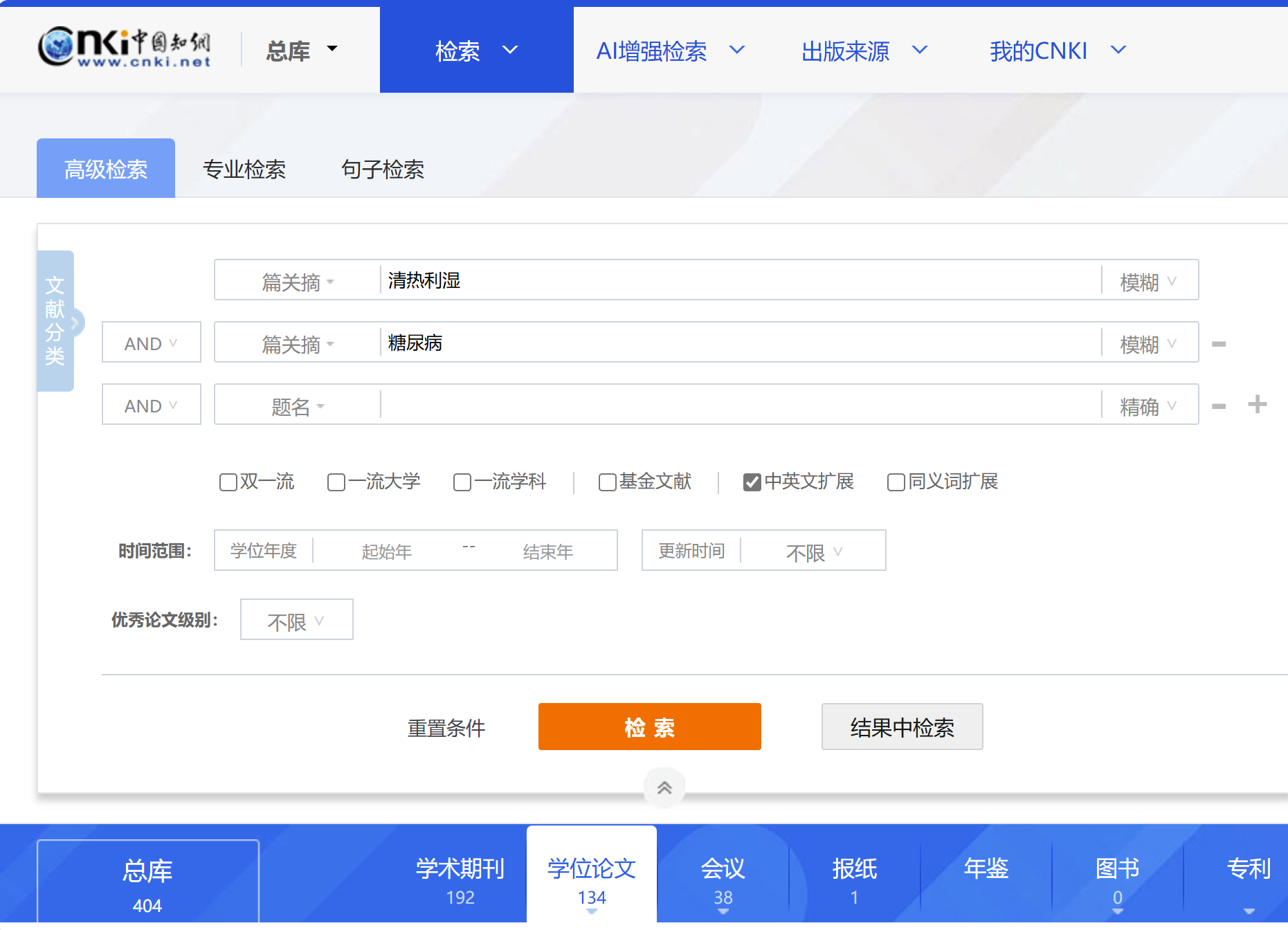

Supplement: Supplementary File 1 — Search strategy. PRISMA Checklist. [file Supplementaryfile1.doc]
